# A comparative study on two methods of ocular surface microbial sampling

**DOI:** 10.1186/s12886-023-02979-1

**Published:** 2023-05-22

**Authors:** Xinyi Shen, Yi Xu, Jinzhi Huang, Peiyu Wu, Weihe Zhou, Yanyan Chen

**Affiliations:** National Clinical Research Center for Ocular Diseases, Eye Hospital, Wenzhou Medical University, Wenzhou, 325027 China

**Keywords:** Cornea, Infection surveillance and control, Microbial aerosol, Sampling method, COVID-19, Ocular surface microbial infection, Conjunctival sac

## Abstract

**Purpose:**

To compare the effect of traditional conjunctival sac swab sampling (A) with aerosolization ocular surface microorganism sampling (B),a novel microbial sampling method, in detecting ocular microbial infection.

**Methods:**

The study included 61 participants (122 eyes) enrolled at the Eye Hospital, Wenzhou Medical University from December, 2021 to March, 2023. Each eye of the participants underwent sampling first with method A then B.Before aerosolization sampling, the air environment was disinfected and sampled as blank air control sample. Subsequently, the air pulses impinging the ocular surface causes dehiscence of the tear film covering the ocular surface and aerosols are formed.The microorganisms from the ocular surface attach to the aerosols generated as aerosolization ocular surface microorganism and be sampled as subject sample by bio-aerosol sampler.The samples were collected and incubated at 25℃ for 3–5 days and 37℃ for 24–48 h.The colonies were counted and identified by matrix-assisted laser desorption/ionization time-of-flight mass spectrometry.

**Results:**

The accuracy in Group B was higher than that in Group A (45.8% vs. 38.3%, P = 0.289). There was a slight level of agreement between the results from both the sampling methods (k = 0.031, P = 0.730). The sensitivity in Group B was higher than that in Group A (57.1% vs. 35.7%, P = 0.453). The specificity results in Group B was higher than that in Group A (44.3% vs. 38.7%, P = 0.480). There were 12 and 37 types of microbes detected in Groups A and B, respectively.

**Conclusions:**

Compared with traditional swab sampling, the novel aerosolization sampling method shows higher accuracy and more comprehensive detection of microbes; however, it cannot completely replace swab sampling. The novel method can be a novel conducive strategy and supplement swab sampling to auxiliary diagnose ocular surface infection.

**Supplementary Information:**

The online version contains supplementary material available at 10.1186/s12886-023-02979-1.

## Introduction

Eye infection can be caused by bacteria, fungi, viruses and parasites. Microorganisms exist in the ocular mucosa, and bacteria are the major contributor of ocular infections worldwide [1–4]. Bacteria are generally associated with many types of ocular infections such as conjunctivitis, keratitis, endophthalmitis, blepharitis,orbital cellulitis and dacryocystitis manifestations [5]. Corneal diseases are still the second most important cause of blindness globally in the world. Their epidemiology is very complex, including a variety of infectious and inflam- matory eye diseases. The eye acts as a portal of entry for SARS-CoV-2 to infect respiratory cells or viral shedding from respiratory cells via the nasolacrimal duct unto the ocular surface. The possibility of ocular secretions as a source for external spread of SARS-CoV-2 has substantial public health implications [6]. The rapid and accurate detection of pathogenic microorganisms in the early stage of eye infection directly affects the effect of disease diagnosis and treatment. It is important to improve the accuracy of the detection and treatment of ocular pathogens.Conjunctival sac swab sampling method is an important method to detect microorganisms on the ocular surface at present, but this method has limited contact area with the ocular conjunctiva surface and is easy to be polluted by microorganisms on the eyelids, eyelashes and surrounding skin which leads to sample pollution and misdiagnosis; At the same time, the conjunctival sac swab sampling method needs to smear the collected swab on the blood agar for inoculation, and rely on the manual operation of the inspectors for bacterial inoculation. Due to the influence of many factors, the test results cannot be standardized, and the efficiency and accuracy of the test results are affected [7, 8]. This process will reduce the microbial samples collected in the conjunctival sac, which is easy to cause missed diagnosis. At present, many studies have shown that the positive rate of conjunctival sampling, inoculation and culture is approximately 50% [9–12]. There are some references of this technique being used in body fluid detection.Instead of cumbersome, time consuming, and expensive progress in swab sampling such as preprocessing, some studies have aerosolized liquid from human oral cavity, the unmodified biological samples, which are sampled and identified rapidly. The operation is simple, efficient, and direct [13, 14].

Therefore, we attempts to propose a new sampling method to improve the accuracy of the test, in order to compare and analyze its clinical application effect with the conventional conjunctival sac swab sampling method.

## Materials and methods

### Study subjects

This experiment included 122 eyes of 61 participants enrolled at the Eye Hospital, Wenzhou Medical University from December, 2021 to March, 2023.The inclusion criteria were as follows: (a). no COVID-19 (the results of novel coronavirus nucleic acid test are negative); (b). able to cooperate with eye examinations.The exclusion criteria were as follows:(a).contact lens wears.(b).ocular surface sampling is not accepted.This study followed the tenets of the Declaration of Helsinki and was approved by the Ethical Committee of The Eye Hospital, Wenzhou Medical University, China (Application number: 2020-018-K-16). Before entering the study cohort, informed consent was obtained from all subjects.

### Specimen collection

#### Preparation before experiments

**Fig. 1:**
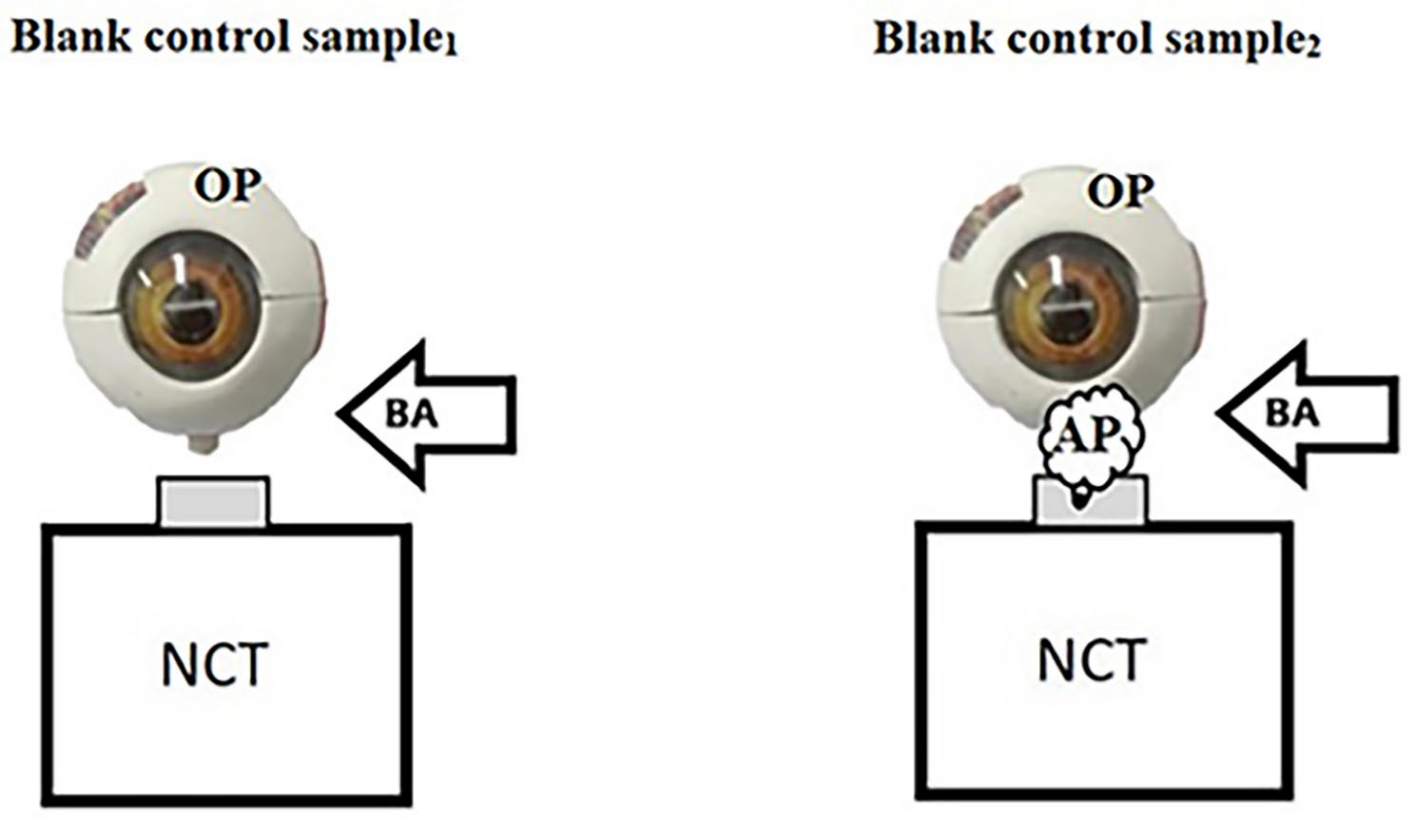
Blank control groups. BA represents the bio-aerosols sampler. OP represents the ophthalmophantom.AP represents air pulses

Environmental preparation:Before conducting the experiments, the room was ventilated and cleaned.Moreover,the object surfaces,air and instruments were disinfected using 75% alcohol to decrease bio-aerosols at the baseline.Air ideal® 3P sampler (BioMérieux) was used to sample initial blank air control samples to obtain the baseline level of bio-aerosols in air and air jet from the non-contact tonometer (TX-20 Automatic NCT, Canon Co, Japan) before the human eyes were aerosolized. Air ideal® 3P technology is based on impacting particles from an airstream onto an agar surface, which was set to allow an air volume of 100 L/min. The device was regulated to collect 30 L of samples marked for blank control sample_1_ (Fig. 1).

Subsequently, NCT was switched on to produce air pulses to flatten the ophthalmophantom (Fig. 1). Meanwhile, Air ideal® 3P was used to collect 30 L of blank control sample_2_, which was set to allow an air volume of 100 L/min. When the blank control sample_1_ and blank control sample_2_ were sampled, the sampler was placed in the direction perpendicular to the air flow at the air jet port.

To maintain the stability of air flow during the trial, unnecessary personnel movement along with frequent door opening and closing were avoided. During experiments, the ambient temperature was maintained between 22 and 28 °C, and the humidity was maintained 45-60%. The room area was approximately 30 m^2^.

Instruments preparation:We sterilized the NCT,bio-aerosols sampler with 75% alcohol.In the tested samplers, we used the blood agar plates (Bio-kont, China) with a diameter of 90 mm.Air ideal® 3P sampler (BioMérieux) was used to collect aerosol samples.The same bio-aerosols sampler was used throughout the experiment.

Personnel preparation:The experiments were conducted under the same indoor personnel density, with only one participant present in the experimental room during the measurement.All the experimenters had undergone standard training.The experimenter wore surgical masks, goggles, work clothes, and sterile hats during the experiments.The participants were required to wear a surgical mask to reduce the unrelated interferences (microbes and aerosols from participant’s mouth and nose) which disturb sampling.

#### Specimen collection in Group A and Group B

Both methods,swab sampling (A) and aerosolization sampling (B) were performed on all eyes; method A was first used for sampling.

### Conventional conjunctival sac swab sampling method (Group A)

**Fig. 2:**
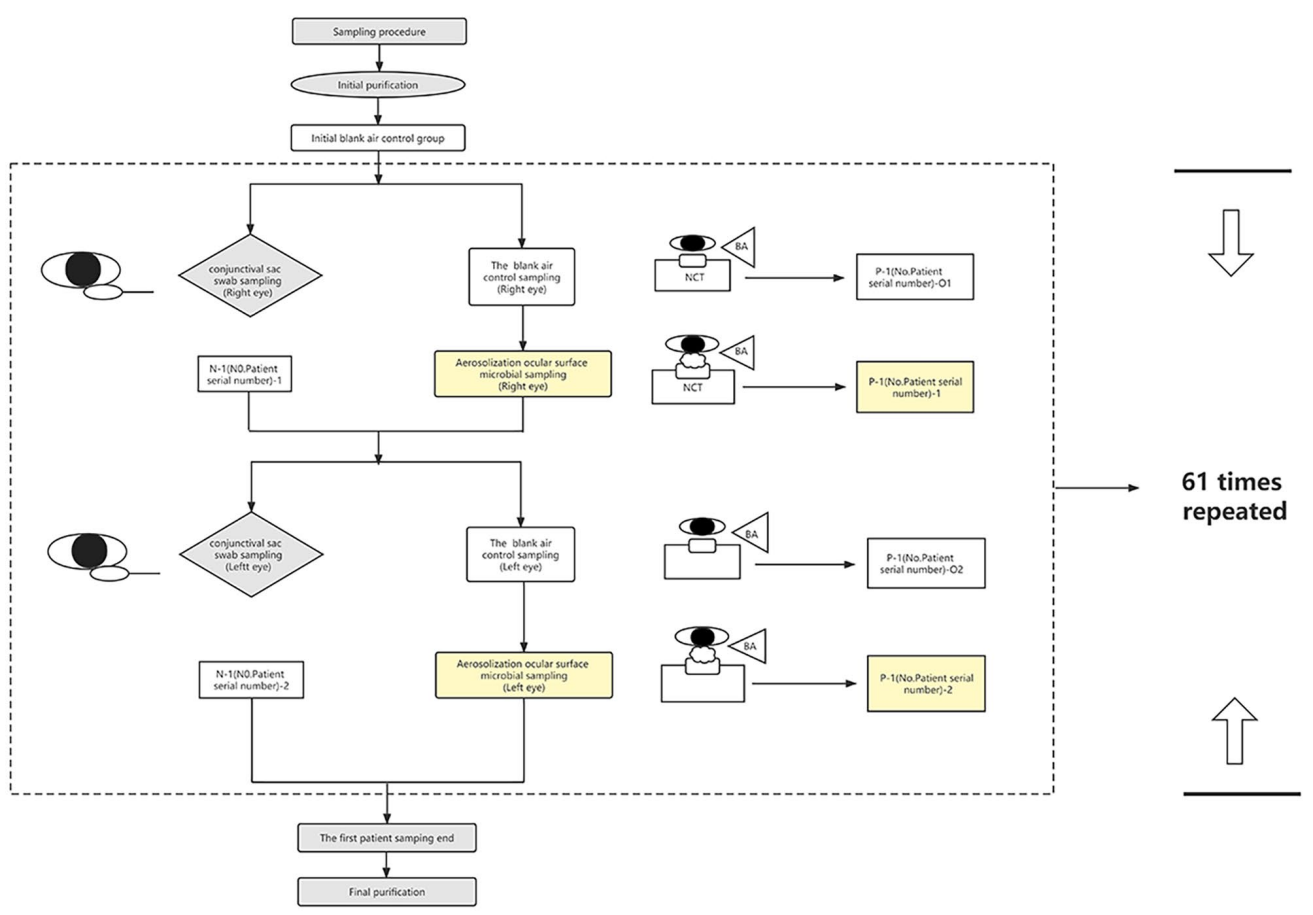
Schematic diagram of aerosolization sampling procedure. BA represents the bio-aerosols sampler.P depicts the samples collected by aerosolization ocular surface microorganism sampling.N depicts the samples collected by conventional conjunctival sac swab sampling method.The first digit after P/N represents the patient number, while the second represents the right (1)/ left (2) eyes. O1 stands for blank air control sample before the right eye; O2 stands for blank air control sample of the left eye

Samples were collected by professionals from the conjunctival sac of each eye of every individual who were in the same batch of patients as who accepted aerosolization sampling. Before sampling, the professional moistened the swabs with sterile normal saline. No anaesthetic was used during the sampling. Before the procedure, we provided an explanation for participants. The participants were required to seat, wear masks, and remain silent throughout the process. They were requested to look up, following which their lower eyelids were turned over, which exposed the lower bulbar conjunctiva and lower fornix conjunctiva. The professional placed the swab containing sterile normal saline on the inner canthus and rotated outwards. The professional gently wiped the surface of the lower conjunctival sac and lower eyelid conjunctiva (while preventing the disregarded of the inner canthus), besides avoiding touch the eyelashes and eyelid margin. Following sampling, the professional transferred the secretion on blood agar, and labelled it as swab sample N_1_ (Fig. 2).

### Aerosolization ocular surface microorganism sampling method (Group B)

**Fig. 3:**
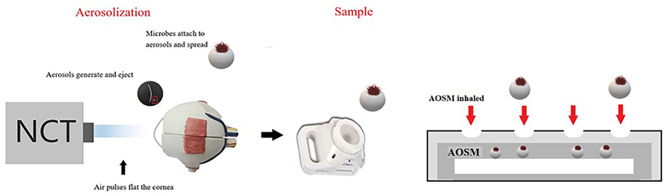
Aerosolization ocular surface microorganism sampling method. AOSM:Aerosolization ocular surface microorganism

Aerosolization ocular surface microorganism sampling means that the eyes of the examinee opened naturally and the ocular surface exposed adequately.A brief, controlled pulse of air is used to impinge the ocular surface which is covered by the tear film (Fig. 3,Video 1).Impingement of the air puff leads to tear film deformation, dehiscence, and aerosols formation and liberation(Fig. 3,Video 1).The microorganisms originating from the ocular surface attach to the aerosols; thus, aerosolization ocular surface microorganism are generated and dispersed; following which the aerosols containing human microorganisms are sampled for identification (Fig. 3,Video 1).

The same batch of patients as the swab sample were enrolled.

Samples from air before aerosolization (Blank air control samples): Before each eye was aerosolized by the novel sampling method, the surrounding air and NCT were disinfected using 75% alcohol.Participants were requested to maintain a seated posture, wear surgical mask, and remain silent. Air ideal® 3P was used to collect 30 L of air sample marked as P-1-O_1_, which was set as the blank air control group (to obtain the baseline level of bio-aerosols in air before each eye was aerosolized, to ascertain and retain the truly present bio-aerosols from the target human eyes, and to eliminate interference factors such as oral and nasal exhalations of the patient and examiner) to control the human eyes aerosolization as a unique variable. The results were compared to the samples obtained from the human eyes during aerosolization (Fig. 2).

Samples from human eyes during aerosolization (Subjects samples):After P-1-O_1_ sampled, we sampled P-1-1. Subjects were required to maintain a seated posture, wear surgical mask, and remain silent during aerosolization.They kept their chin fixed on the chin rest of the NCT, focused on the light source in the jet port, opened their eyes naturally and exposed the ocular surface.We used NCT to produce air pulses to aerosolize the ocular surface microorganisms by impacting the ocular surface leading to tear film dehiscence and aerosol formation. Subsequently, the aerosolized ocular surface microorganisms were sampled by Air ideal® 3P during each eye aerosolization.

The resulting air streams containing bio-aerosols were configured to let through an air volume of 100 L/min.The generated, aerosolized ocular surface microorganisms were captured and directed onto the agar surface which was embedded in the bio-aerosols sampler.There was 30 L of bio-aerosols sampled from each eye.During the experiment, the bio-aerosols sampler was set in the direction perpendicular to the air flow and positioned horizontally next to the air jet of the NCT outside the eyes for air sampling and fixed on the connecting line between the air jet and ocular surface leaning towards the side of the tear film (Fig. 2). After collection, the samples were marked as P-1-1 (Fig. 2). For testing each eye, we repeated 61 times to test 61 participants (Fig. 2).

Further, to avoid the potential effect of order of testing for each participant,the testing order was as follows (Fig. 2): (1) The right eye accepted conventional conjunctival sac swab sampling (Method A) (2) The right eye accepted aerosolization ocular surface microorganism sampling method(Method B) (3) The left eye accepted conventional conjunctival sac swab sampling (4) The left eye accepted aerosolization ocular surface microorganism sampling.

### Cultivation and identification

The plates were incubated at 25˚C in the same an aerobic chamber for 3–5 days and 37˚C in the same an aerobic chamber for 24–48 h after sample collection and the total colony counts were obtained.We corrected the microbial colonies cultured according to the table matched with the instrument.The species of each colony were identified using the matrix-assisted laser desorption/ ionization time-of -flight mass spectrometry (MALDI TOF MS).In this experiment, we performed a double-blind study,the experimental sampling personnel are different from the staffs for identification.

### Statistical analysis

EpiData 3.1 is used to establish a database for parallel double input, and statistical analyses were performed using IBM SPSS Statistics for Windows (version 25.0; IBM Corp., Armonk, NY,USA).Quantitative data were expressed as mean ± standard deviation or median, interquartile range. Qualitative data were expressed as percentages.McNemer test was used for comparison between groups.The Shapiro-Wilk test indicated that the evaluation indicators in this study were nonnormally distributed.When using the volumetric method, CFUs were calculated using the correction table associated with the equipment.The colonies were identified by matrix-assisted laser desorption/ionization time-of-flight mass spectrometry (MALDI-TOF-MS). Microbial density was measured as colony-forming units per plate (CFU/plate).The gold standard is whether patients are symptomatic or asymptomatic, and whether or not there was clinical evidence of ocular surface infection (the final medical diagnosis entered in the hospital system after a professional ophthalmologist from a first class hospital diagnosed). P < 0.05 indicated statistical significance.

## Results

The mean age of the participants in the study was 38.91 ± 15.46 years.Twenty seven (44.26%) of the participants were male and 34 (55.74%) were female.

### Comparison of accuracy

The accuracy in Group B was higher than that in Group A, the difference was not statistically significant (45.8% vs. 38.3%, P = 0.289)(Table 1).There was a slight level of agreement between results from both sampling methods, the agreement was not statistically significant (k = 0.031, P = 0.730).

**Table 1:** Comparison of the accuracy of Groups A and B

		Group B		
Group A	＋		-	Total
＋	22		24	46
-	33		41	74
Total	55		65	120

Note:Two plates in Group B were missed completely at random because they were contaminated and lost accidentally.Finally,The number of samples included was 120 in Group A and Group B,respectively

The sensitivity in Group B was higher than that in Group A (57.1% vs. 35.7%, P = 0.453)(Tables 2, 3 and 4).There was a slight level of agreement between results from both sampling methods,the agreement wasn’t statistically significant (k = 0.039, P = 0.872).

**Table 2:** Comparison of the detection results of gold standard and Group A

		Gold standard		
Group A	＋		-	Total
＋	5		65	70
-	9		41	50
Total	14		106	120

Note: The gold standard is patients symptomatic or asymptomatic,and they whether or not had clinical evidence of ocular surface infection ( the final medical diagnosis entered in the hospital system after professional ophthalmologist from a first class hospital diagnosed

The specificity results in Group B was higher than that in Group A (44.3% vs. 38.7%, P = 0.480)(Tables 2, 3 and 5).There was a slight level of agreement between results from both sampling methods,the agreement wasn’t statistically significant (k = 0.032,P = 0.742).

**Table 3:** Comparison of the detection results of gold standard and Group B

		Gold standard		
Group B	＋		-	Total
＋	8		59	67
-	6		47	53
Total	14		106	120

**Table 4:** Comparison of the sensitivity results of Groups A and B

		Group A		
Group B	＋		-	Total
＋	3		5	8
-	2		4	6
Total	5		9	14

**Table 5:** Comparison of the specificity results of Groups A and B

		Group A		
Group B	＋		-	Total
＋	37		22	59
-	28		19	47
Total	65		41	106

The number of positive samples in Group B was lower than that in Group A, with 67 (55.8%) positive results in Group B and 70 (58.3%) positive results in group A.The positive rate of colony detection in Group B was lower than that in Group A (P = 0.791)(Table 6).There was a slight level of agreement between results from both sampling methods,the agreement wasn’t statistically significant(k = 0.031,P = 0.733).

**Table 6:** Comparison of the detection results of Groups A and B

		Group A		
Group B	＋		-	Total
＋	40		27	67
-	30		23	53
Total	70		50	120

### Comparison of comprehensive

**Fig. 4:**
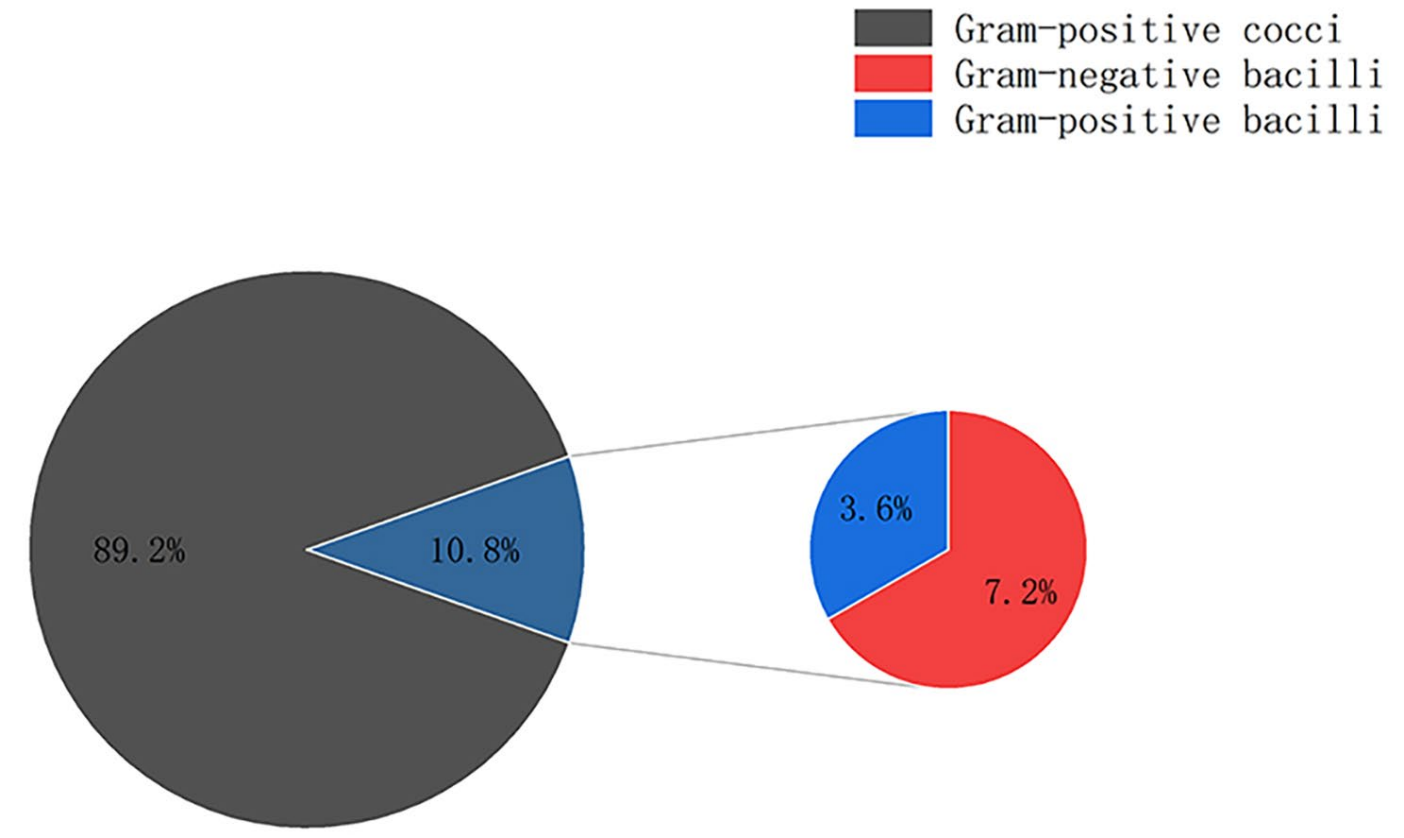
Pie chart showing Gram’s staining findings of Group A

**Fig. 5:**
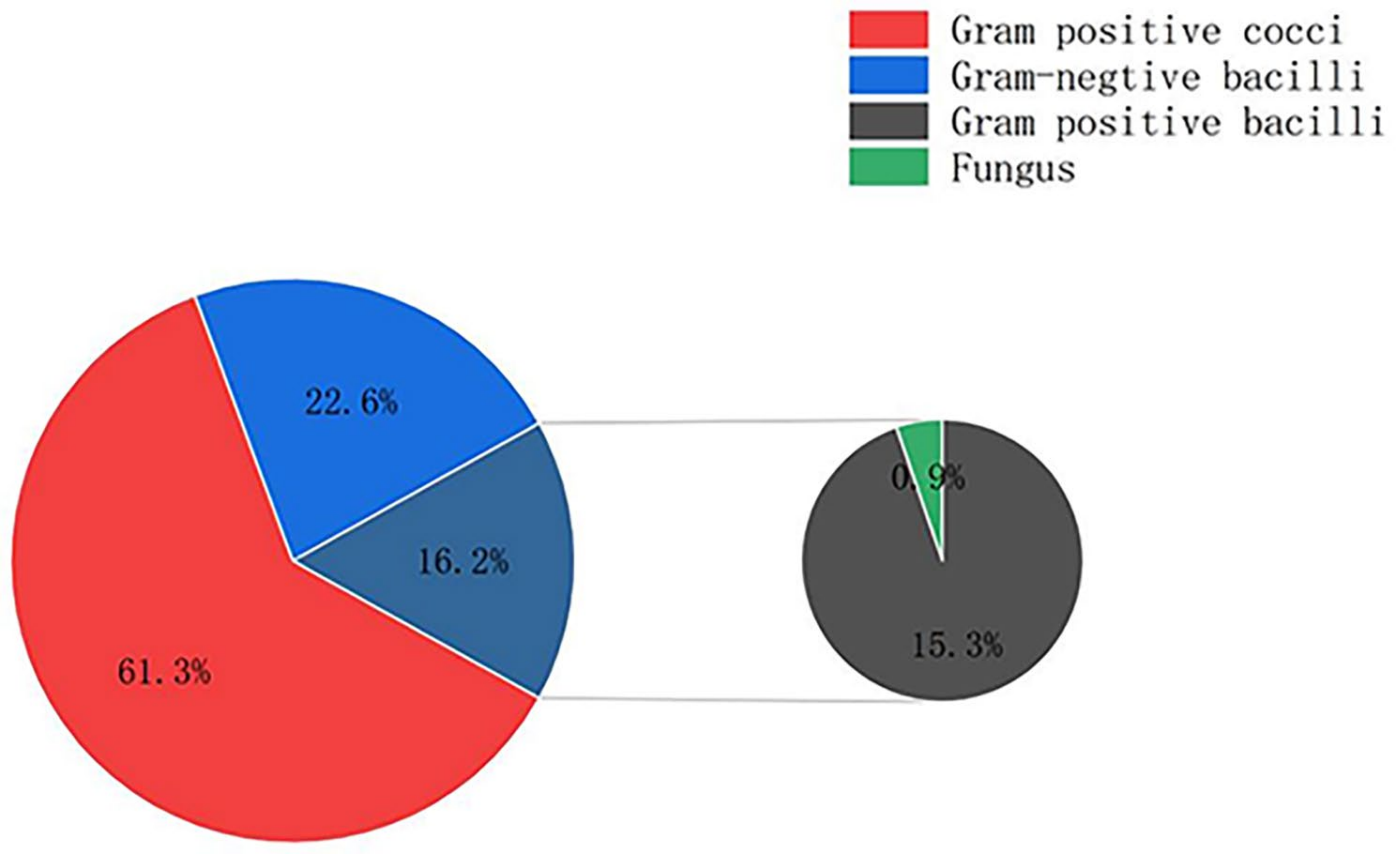
Pie chart showing Gram’s staining findings of Group B

A total of 12 types of microbes were detected in Group A and 37 types of microbes were detected in Group B (Figs. 4 and 5; Table 7).

**Table 7:** Number and proportion of bio-aerosol species detected by novel method

Bio-aerosols species	Number of samples	Proportion
*Staphylococcus epidermidis*	34	13.93%
*Kocuria marina*	16	6.56%
*Kocuria rhizophila*	14	5.74%
*Bacillus cereus*	7	2.87%
*Kocuria palustris*	13	5.33%
*Bacillus megaterium*	5	2.05%
*Kocuria*	6	2.46%
*Micrococcus antarcticus*	5	2.05%
*Staphylococcus warneri*	9	3.69%
*Moraxella sg Moraxella osloensis*	7	2.87%
*Rhizobium radiobacter*	4	1.64%
*Micrococcus endophyticus*	6	2.46%
*Rothia endophytica*	3	1.23%
*Staphylococcus equorum*	3	1.23%
*Staphylococcus hominis*	13	5.33%
*Bacillus*	9	3.69%
*Bacillus infantis*	5	2.05%
*Lactococcus*	4	1.64%
*Exiguobacterium aurantiacum*	6	2.46%
*Aerococcus viridans*	6	2.46%
*Arthrobacter oxydans*	3	1.23%
*Kocuria rosea*	7	2.87%
*Acinetobacter lwoffii*	2	0.82%
*Bacillus subterraneus*	2	0.82%
*Moraxella*	2	0.82%
*Enterobacter hormaechei*	4	1.64%
*Sporosarcina*	2	0.82%
*Stenotrophomonas acidaminiphila*	13	5.33%
*Rothia amarae*	4	1.64%
*Brevundimonas*	4	1.64%
*Staphylococcus saprophyticus*	3	1.23%
*Kocuria sediminis*	3	1.23%
*Aureobasidium pullulans*	2	0.82%
*Burkholderia cenocepacia*	2	0.82%
*Psychrobacter pulmonis*	2	0.82%
*Pseudomonas otitidis*	2	0.82%
*Psychrobacter faecalis*	3	1.23%

Note: The proportion of bio-aerosol samples = Number of samples included in the type of bio-aerosols / Total number of aerosol samples.Two plates in Group B were missed completely at random because they were contaminated and lost accidentally. Total number of aerosol samples was 244(242 samples in Group B + 2 initial blank air control samples)

In Group A, gram-positive cocci accounted for 89.2%,followed by gram-negative bacilli (7.2%), gram-positive bacilli (3.6%)(Fig. 4).Gram-positive cocci were mainly *Staphylococcus* (98.6%), mostly *Staphylococcus epidermidis* (82.4%). *Staphylococcus epidermidis* were detected in 61 of 122 swab samples (50%).

In Group B,gram-positive cocci accounted for 61.3%, followed by gram-negative bacilli (22.6%), gram-positive bacilli (15.3%), and fungi (0.9%)(Fig. 5).The analysis of aerosolized microbes from ocular surface in Group B was shown in Table 8. *Staphylococcus spp.* were detected in 62 aerosolization samples and formed the largest proportion of gram-positive cocci, followed by *Kocuria* (Table 8). The largest proportion of gram-negative bacilli was made up of *S. acidaminiphila* (5.33%)(Tables 7 and 8). Twenty six samples showed the presence of *Bacillus* during aerosolization, accounting for the largest proportion of gram-positive bacilli. *B. cenocepacia* and *A. pullulans* were detected in two samples (0.82%) each (Tables 7 and 8).

**Table 8:** Types and number of bio-aerosols detected by novel method

Name	n		n		n		n
**Gram positive** **cocci**	144						
*Staphylococcus*	62	*Staphylococcus epidermidis*	34	*Micrococcus*	11	*Micrococcus endophyticus*	6
		*Staphylococcus saprophyticus*	3			*Micrococcus antarcticus*	5
		*Staphylococcus warneri*	9				
		*Staphylococcus equorum*	3	*Kocuria*	59	*Kocuria* *palustris*	13
		*Staphylococcus hominis*	13			*Kocuria rhizophila*	14
						*Kocuria* *marina*	16
*Lactococcus*	4	*Lactococcus*	4			*Kocuria sediminis*	3
*Aerococcus*	6	*Aerococcus viridans*	6			*Kocuria rosea*	7
						*Kocuria*	6
*Sporosarcina*	2	*Sporosarcina*	2				
**Gram negative** **bacilli**	**53**						
*Pseudomonas*	2	*Pseudomonas otitidis*	2	*Acinetobacter*	2	*Acinetobacter lwoffii*	2
*Moraxella*	9	*Moraxella sg Moraxella osloensis*	7	*Exiguobacterium*	6	*Exiguobacterium aurantiacum*	6
		*Moraxella*	2	*Enterobacter*	4	*Enterobacter hormaechei*	4
*Brevundimonas*	4	*Brevundimonas*	4	*Rhizobium*	4	*Rhizobium radiobacter*	4
*Burkholderia*	2	*Burkholderia cenocepacia*	2	*Stenotrophomonas*	13	*Stenotrophomonas acidaminiphila*	13
*Psychrobacter*	5	*Psychrobacter pulmonis*	2	*Bacillus*	2	*Bacillus subterraneus*	2
		*Psychrobacter faecalis*	3				
**Gram positive bacilli**	**36**						
*Arthrobacter*	3	*Arthrobacter* *oxydans*	3	*Bacillus*	26	*Bacillus cereus*	7
						*Bacillus megaterium*	5
*Rothia*	7	*Rothia amarae*	4			*Bacillus*	9
		*Rothia endophytica*	3			*Bacillus infantis*	5
**Fungus**	**2**						
*Aureobasidium*	2	*Aureobasidium pullulans*	2				

Note: n is the number of samples in which the bio-aerosol was detected

## Discussion

### Aerosolization sampling has higher accuracy

The accuracy in Group B was higher than that in Group A,the difference wasn’t statistically significant (45.8% vs. 38.3%,P = 0.289)(Table 1).There was a slight level of agreement between results from both sampling methods,the agreement wasn’t statistically significant (k = 0.031,P = 0.730).The sensitivity in Group B was higher than that in Group A (57.1% vs. 35.7%, P = 0.453)(Tables 2, 3 and 4).The specificity results in Group B was higher than that in Group A (44.3% vs. 38.7%,P = 0.480)(Tables 2, 3 and 5).There wasn’t statistically significant difference between the positive rate of A and B (58.3% vs. 55.8%,P = 0.791).

Swab sampling method, is wetted by sterile normal saline or TSB with sterile swab, and then coated and inoculated on the surface of blood agar plate by rolling with cotton swab indirectly [15]. The results can be contaminated from the laboratory environment,personnel and during sampling [8, 16]. So,it is easy to leave and mixed with contaminated bacteria in the indirect inoculation process, resulting in misdiagnosis.The results show that the accuracy of aerosolization sampling method are higher.We speculate that the reason may be that the bio-aerosols directly fall on the Petri dish, eliminating the intermediate artificial inoculation link, and collecting the ocular surface colonies more comprehensively, which improves the accuracy rate and reduces the rate of missed diagnosis and misdiagnosis.

There are some results (positive or negative) detected in both Groups A and B which were different from the gold standard.The reason for this phenomenon is that the final medical diagnosis is after professional ophthalmologist combines the bacteria results with the patient’s clinical situation and other factors synthetically.As a good sampling method needs to provide comprehensive microbes results from the ocular surface for judgement.

### Aerosolization samples more comprehensively

*Staphylococcus epidermidis* was detected with the highest proportion in both Groups A and B,which were detected in 61 of 122 swab samples (50%) and 34 of 244 aerosol samples(13.93%)(Table 7).In Group A, gram-positive cocci were mainly *Staphylococcus* (98.6%), mostly *Staphylococcus epidermidis* (82.4%);In Group B,*Staphylococcus spp.* was detected in 62 aerosolization samples and formed the largest proportion of gram-positive cocci, followed by *Kocuria.*The composition of bio-aerosol was concordant with the distribution of microbes on the ocular surface, and mainly comprised *Staphylococcus*[10].

Besides,among 37 types of bio-aerosols were detected in Group B, gram-positive cocci accounted for 61.3%, followed by gram-negative bacilli (22.6%), gram-positive bacilli (15.3%), and fungi (0.9%).The composition was similar to those in Group A that gram-positive cocci accounted for 89.2%,followed by gram-negative bacilli (7.2%),gram-positive bacilli (3.6%).Our results suggest that the microbes truly present on the ocular surface can be aerosolized and sampled by the novel aerosolization sampling method.

Further more,compared with the traditional swab sampling method, aerosolization sampling method collected more abundant type of bacteria.

In our results, a total of 12 and 37 types of microbes were detected in Groups A and B, respectively. The largest proportion of gram-negative bacilli was made up of *S. acidaminiphila* (5.33%).This pathogenic microorganism is multidrug resistant and can cause fatal infections in humans [17]. Twenty six aerosolization samples showed the presence of *Bacillus*, accounting for the largest proportion of gram-positive bacilli. *B. cenocepacia* and *(A) pullulans* were detected in two samples (0.82%) each. *(B) cenocepacia* is an important pathogenic bacterium which causes nosocomial infections.It causes diseases such as cystic fibrosis and chronic granulomatous. *Burkholderia, Chryseobacterium*, and *Enterobacteriaceae* have been reported to predominate the lung microbiota in fatal cases of SARS-CoV-2 infection [18]. *Moraxella, Pseudomonas, Bacillus*, and other pathogens also detected in the aerosolization sampling.

Reviewing the domestic large sample research,our results were similar to the domestic reports based on traditional culture, isolation and identification methods: The common pathogenic bacteria on the ocular surface are *Staphylococcus, Streptococcus, Neisseria, Pseudomonas, Moraxella, Enterobacteriaceae, Corynebacterium and Clostridium*[19, 20].

Our results showed that not only can the common microbes distributed on the ocular surface be carried with aerosols by aerosolization,but also some pathogenic microorganisms can also be aerosolized and sampled.We speculate that the possible reason could be that the traditional swab sampling method collect specimens only from a specific site.However,with aerosolization sampling, and the air-pulse impact and aerosolize the tear film in the center of the cornea, which has a larger contact area with the ocular surface.Various microbes distributed widely on the ocular surface were all carried on aerosols during aerosolization and sampled.It showed greater accuracy and more comprehensive detection of bacteria on ocular surface.

Moreover,the detection based on MALDI-TOF-MS makes the diagnosis of ocular pathogens more comprehensive.The technology can more comprehensively reflect the distribution of microorganisms on the ocular surface, effectively improve the accuracy rate,collect the ocular surface colonies more comprehensively, reduce missed diagnosis and misdiagnosis, and is of great significance for the auxiliary diagnosis of ocular surface infectious diseases.

In this study, compared with the traditional conjunctival sac swab, aerosolization ocular surface microorganisms sampling method showed greater accuracy and more comprehensive detection of bacteria on ocular surface.It can effectively avoid the influence of error,and improve the efficiency and accuracy rate.However, it cannot completely replace swab sampling.The novel method can be a novel conducive strategy and a supplement to swab sampling for auxiliary diagnosis of ocular surface infection.It can provide more comprehensive auxiliary reference for professional ophthalmologist to make accurate clinical diagnoses,thereby reducing the missed diagnosis and misdiagnosis rate of ocular surface infectious diseases.

The novel sampling method can provide more time-saving,cost-saving and convenient service for patients.

Future studies will need to increase the sample size for further validation.We are independently developing an integrated device to aerosolization ocular surface microorganisms to replace NCT by generating air puff impacting tear film on ocular surface and sampling of ocular surface microorganisms to replace bio-aerosols sampler for further experiments. Li et al. examined aerosol samples collected by a microbial air sampler, and environmental surfaces were sampled using sterile premoistened swabs at multiple sites. All aerosol samples were negative for SARS-CoV-2[21]. Future research should focus on extracting viruses from suspected infected samples and seeking targeted protective measures.

## Electronic supplementary material

Below is the link to the electronic supplementary material.

Supplementary Material 1

Supplementary Material 2

## Data Availability

The datasets generated and analyzed during the current study are not publicly available due to ethical restrictions but are available from the corresponding author on reasonable request.
