# Supplementary material for: A comparative study on two methods of ocular surface microbial sampling
Source: BMC Ophthalmol. 2023 May 22;23:228. doi: 10.1186/s12886-023-02979-1 (PMC10201025; doi:10.1186/s12886-023-02979-1)
Supplement: Supplementary file 2 — Supplementary Material 2 [file 12886_2023_2979_MOESM2_ESM.docx]

# VIDEO LEGEND

1.The video of Aerosolization ocular surface microorganism sampling

AOSM:Aerosolization ocular surface microorganism;NCT:non-contact tomometer.
